# Causal effects of various types of physical activities on psychiatric disorders: a Mendelian randomization study

**DOI:** 10.3389/fspor.2024.1331586

**Published:** 2024-03-04

**Authors:** Lan Yu, Xu Zhang, Wangshu Li

**Affiliations:** ^1^ Department of Gynaecology, Dalian Women and Children’s Medical Center (Group), Dalian, Liaoning, China; ^2^ Department of Clinical Medicine, Harbin Medical University, Harbin, Heilongjiang, China; ^3^ Department of Clinical Medicine, The Second Affiliated Hospital of Harbin Medical University, Harbin, Heilongjiang, China; ^4^ Department of Key Laboratory for Early Diagnosis and Biotherapy of Malignant Tumors in Children and Women in Liaoning Province, Dalian, Liaoning, China

**Keywords:** exercise, types of physical activity, psychiatric disorders, Mendelian randomization, causal effects

## Abstract

**Background:**

Psychiatric disorders (PD) pose a significant burden, with vast prevalence and mortality, inflicting substantial costs on individuals and society. Despite its widespread prevalence, the complex pathogenesis of PD remains elusive, leading to limited and challenging therapeutic development. An emerging risk factor for chronic diseases, prolonged sedentary behavior, contrasts with the therapeutic potential of exercise, regardless of its intensity, for various ailments, including PD. Yet, the diversity in exercise modalities and intensities may offer varied impacts on health. This study, leveraging Mendelian Randomization (MR), seeks to investigate the causal relationship between exercise and PD, aiming to elucidate the optimal exercise modality and intensity for PD mitigation while addressing potential confounders.

**Methods:**

This study employed a Mendelian randomization analysis using the genome-wide association study (GWAS) database to investigate the causal relationship between types of physical activity and psychiatric disorders. Sensitivity analysis was conducted to demonstrate the reliability and robustness of the results.

**Results:**

In the past 4 weeks, engaging in a substantial amount of DIY physical activity was found to have a causal relationship with psychiatric disorders (IVW: OR = 0.228, 95% CI: 0.113–0.461, *P* = 0.000038). As for the types of exercises, there may be a potential causal association between aerobic training (including swimming, cycling, fitness, and bowling) and psychiatric disorders (IVW: OR = 0.322, 95% CI = 0.148–0.704, *P* = 0.004). However, there was no causal relationship found between mild DIY physical activity and psychiatric disorders (IVW: OR = 0.918, 95% CI = 0.417–2.021, *P* = 0.831). Furthermore, it seems that there is no causal relationship between vigorous exercise and psychiatric disorders (IVW: OR = 2.705, 95% CI = 0.081–3.419, *P* = 0.578).

**Conclusion:**

Our study confirms that only a certain level of training activity can have a protective effect on psychiatric disorders, while mild physical activity or vigorous training does not have an impact on psychiatric disorders.

## 1 Introduction

Psychiatric disorders (PD) have been widely acknowledged for their vast prevalence and mortality, exerting a tremendous socio-economic toll (1, 2). Accounting for approximately 7% of the global disease burden, PD affects nearly one billion individuals globally, underscoring its profound human impact (3, 4). The intricacy of PD's onset mechanisms, still largely enigmatic, has rendered the development of treatments for most psychiatric illnesses both limited and intricate (5). Hence, there's an acute demand for innovative therapeutic interventions.

Sedentary behavior, characterized by prolonged periods of low energy expenditure while awake (sitting/reclining), is increasingly recognized as another perilous harbinger for chronic ailments (6, 7). Emerging theories suggest that physical activity influences psychiatric disorders through multiple mechanisms, including the enhancement of brain plasticity, modulation of stress hormones, and improvement in sleep patterns. Exercise is believed to stimulate the release of neurotrophic factors, such as Brain-Derived Neurotrophic Factor (BDNF), which supports neurogenesis and neural health. Additionally, physical activity reduces levels of stress hormones like cortisol, which is often elevated in psychiatric conditions. Furthermore, engaging in exercise improves sleep quality and patterns, which have a direct impact on mental health. These interconnected pathways collectively contribute to the therapeutic effects of physical activity on psychiatric disorders. In stark contrast, physical activity, irrespective of its quantum, has demonstrated therapeutic promise for a spectrum of chronic conditions, encompassing PD (8). Although habitual and moderate-level physical exertion has been conclusively shown to confer benefits against PD in the general populace (9), the sheer diversity in exercise regimens and their associated intensities might elicit profoundly varied physiological responses (10, 11). Thus, pinpointing the exercise regimen and intensity optimally tailored for PD has emerged as a pivotal therapeutic directive, with our understanding in this realm still in its infancy (12). Furthermore, individuals routinely engaging in physical activity often concurrently adopt healthier lifestyle habits such as a nutritious diet, moderated alcohol and tobacco consumption, and a positive life outlook (13), which could potentially confound causal observations by attributing PD improvements solely to exercise. On the flip side, observed associations might stem from a reverse causality scenario where PD influences behavioral patterns, leading to variations in hosts' activity levels. However, discerning their roles in the etiological causation of diseases presents a significant challenge, primarily when both exercise and PD data heavily lean on patients' self-reporting, thereby lacking compelling evidential validity.

Here, we propose harnessing Mendelian Randomization (MR) as a formidable tool to dissect the causal relationship between exercise and PD while concurrently curtailing biases stemming from confounding and reverse causality (14). By capitalizing on genetic variations as instrumental variables for exposure, MR is well-poised to unravel true causal relationships, given the hereditary arbitrariness of genetic sequences that renders associations between genetic variations and outcomes largely immune to common confounders (15). With its proven track record in delineating causal relationships between PD and a plethora of influential factors such as circulatory biomarkers (16), inflammation (17), and antihypertensive medications (18), MR's applicability in ascertaining etiological factors for PD stands validated. In this investigative endeavor, we have leveraged data from the IEU OpenGWAS project to conduct a unidirectional MR analysis, aiming to gauge the causal interplay between various physical activity modalities and PD.

## 2 Materials and methods

### 2.1 Study design

Embracing a two-sample Mendelian Randomization paradigm, this study sourced from genome-wide association study (GWAS) databases, aspired to decode the causal nexus between types of physical activity and psychiatric disorders. Subsequent sensitivity analyses were meticulously conducted to attest to the dependability and steadfastness of the findings. Detailed specifics of the incorporated GWASs databases are elaborated in Table 1. Furthermore, the Mendelian randomization study requires three core assumptions, As presented in Figuer 1, which are: (1) The extracted instrumental variable single nucleotide polymorphism (SNP) must be strongly associated with the exposure; (2) Instrumental variable SNPs should not be associated with any confounders of the “exposure-outcome” relationship; (3) Instrumental variable SNPs can only influence the outcome through the exposure.

**Table 1:** GWAS datasets used in the mendelian randomization study.

Variables	ID	Sample	Race	Year
Psychiatric diseases	finn-b-F5_PSYCH	218,792 (control: 164,296;case: 54,496)	European	2021
Types of physical activity in last 4 weeks: Light DIY (e.g.,: pruning, watering the lawn)	ukb-b-11495	460,376 (control:224,132;case:236,244)	European	2018
Types of physical activity in last 4 weeks: Heavy DIY (e.g.,: weeding, lawn mowing, carpentry, digging)	ukb-b-13184	460,376 (control:263,370; case: 197,006)	European	2018
Types of physical activity in last 4 weeks: Strenuous sports	ukb-b-7663	460,376 (control: 412,908;case: 47,468)	European	2018
Types of physical activity in last 4 weeks: Other exercises (e.g.,: swimming, cycling, keep fit, bowling)	ukb-b-8764	460,376 (control:237,906;case: 222,470)	European	2018

This table lists the GWAS datasets drawn upon in this study, detailing the specific types of physical activities, associated dataset IDs, sample sizes (divided into control and case), population race, and the year of the study.

**Figure 1:**
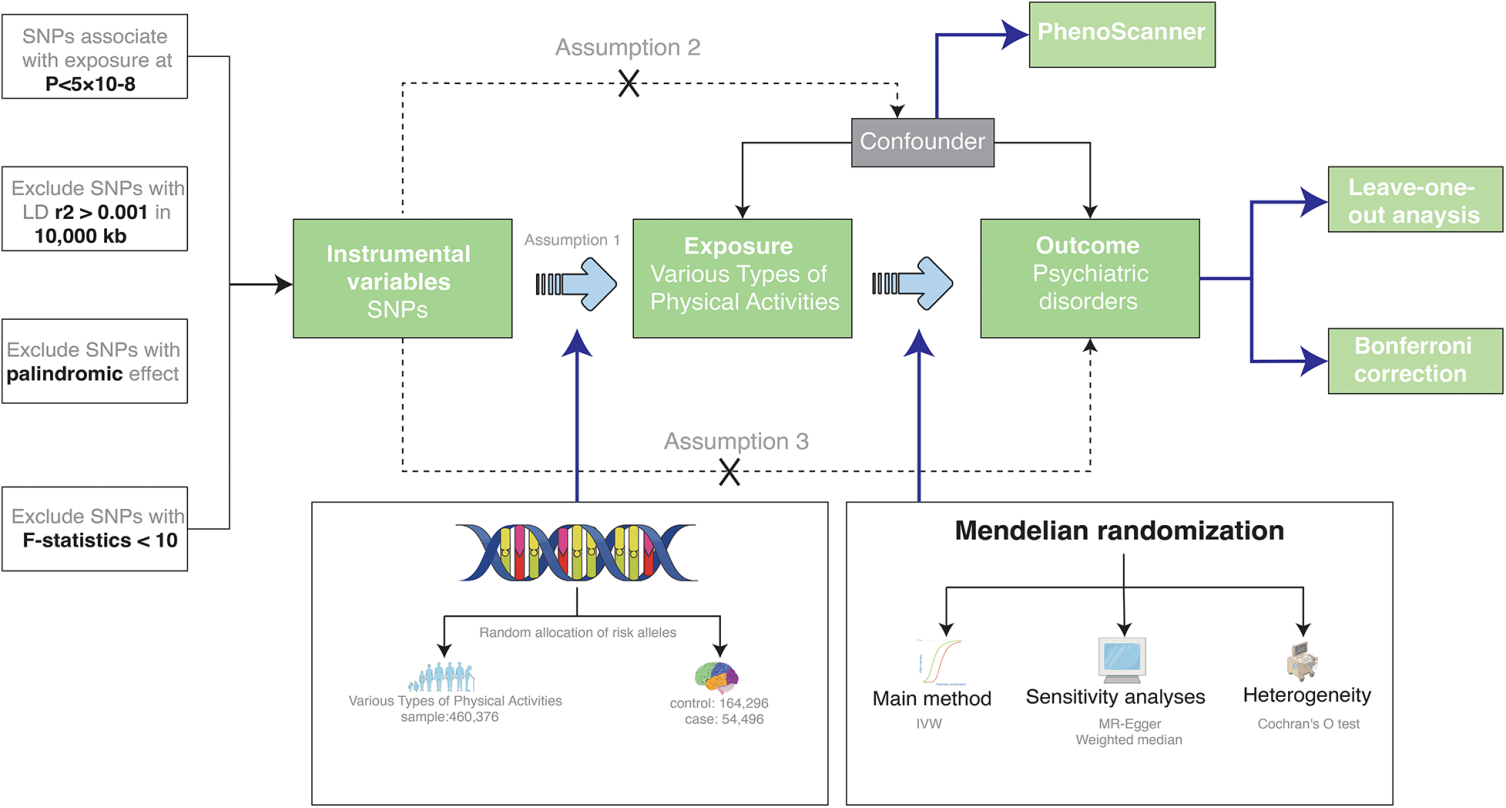
A two-sample MR design. IVW stands for Inverse-Variance Weighted, which is our primary method for examining the association between exposure and outcome. LD represents Linkage Disequilibrium, used to assess the correlations between SNPs, which are Single Nucleotide Polymorphisms. SNPs serve as our genetic instrumental variables for the study. MR refers to Mendelian Randomization, our overarching approach.

Ethical approval was not necessary for this study as it utilized publicly accessible GWAS data. We conducted our research in alignment with the “STrengthening the Reporting of OBservational studies in Epidemiology using Mendelian Randomization (STROBE-MR)” guidelines.

### 2.2 Data source

All GWAS databases incorporated in this article were obtained from the publicly accessible IEU OpenGWAS project (https://gwas.mrcieu.ac.uk/).

### 2.3 Instrumental variable selection

Instrumental variable SNPs were selected in alignment with the three core assumptions of Mendelian randomization previously discussed. Criteria for SNP selection were: *P* < 5 × 10^−8^, linkage disequilibrium *r*^2^ of 0.001, and a linkage disequilibrium region width of 10 Mb. Moreover, to negate the influence of genetic variants with potential confounders, SNPs were cross-referenced in the PhenoScanner database (http://www.phenoscanner.medschl.cam.ac.uk/) to ensure they were not associated with any confounders. Subsequently, SNPs exhibiting significant heterogeneity were excluded based on heterogeneity tests. After the outlined selection process, instrumental variable SNPs associated with psychiatric disorders were identified. The F-statistic was computed for these variables, and those with *F*-values consistently above 10, indicating no weak instrumental variables, were incorporated into the Mendelian randomization analysis.

### 2.4 Mendelian randomization analysis

During the Mendelian randomization analysis, the primary method utilized was the random-effects inverse-variance weighted (IVW) approach. Traditional IVW can be influenced by factors like pleiotropy; thus, complementary methods like MR-Egger and weighted mode were employed for MR analysis. To mitigate biases arising from horizontal pleiotropy, heterogeneity tests, horizontal pleiotropy evaluations, and leave-one-out analyses were concurrently used for sensitivity checks to verify the robustness of outcomes. All statistical examinations employed two-tailed tests, and the results were adjusted using Bonferroni correction to counteract potential biases from multiple data analyses. A *P*-value <0.00125 was considered statistically significant, while results with *P*-values between 0.05 and 0.0125 were deemed to imply a potential causal relationship. In this study, data processing was conducted using the “TwoSampleMR” and “MR-PRESSO” packages in R.

## 3 Results

### 3.1 Instrumental variable

Determination Drawing from the core assumptions of Mendelian randomization outlined earlier, namely *P* < 5 × 10^−8^ and linkage disequilibrium *r*^2^ of 0.001, we excluded potential confounders. After ensuring all F-statistics were greater than 10 to remove weak instrumental variables, 45 SNPs were finalized as instrumental variables in Supplementary Table S1.

### 3.2 Heavy DIY activities and their association with psychiatric disorders

**Figure 2:**
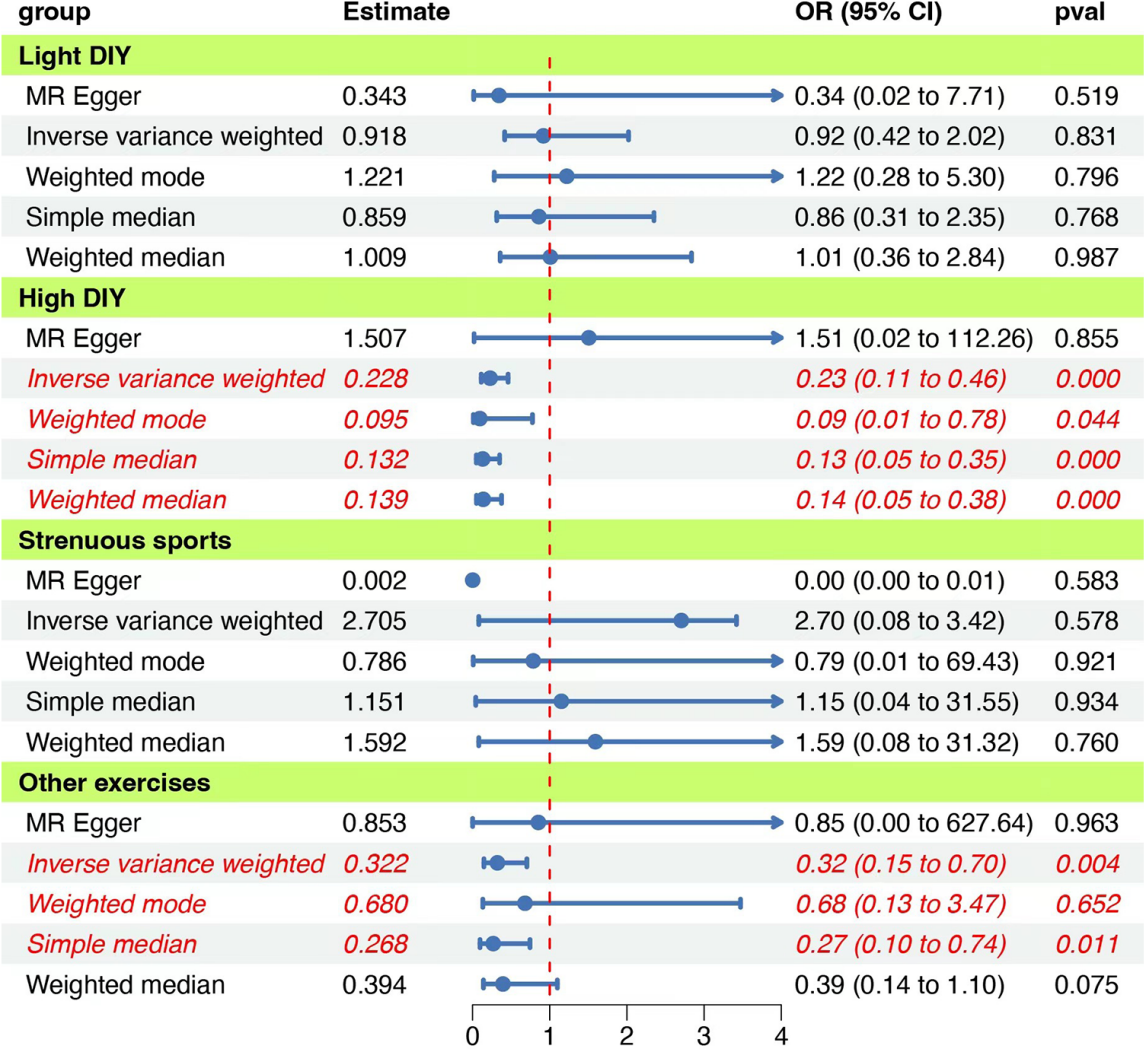
Forest plot of the MR analyses for the associations between physical activities and psychiatric disorders. The figure presents various estimation methods and their corresponding odds ratios (OR) with 95% confidence intervals (CI) for different activity groups, Each group uses methods such as MR Egger, Inverse variance weighted, Weighted mode, Simple median, and Weighted median. The vertical red dashed line represents an OR of 1, serving as a reference point. The “pval” column indicates the statistical significance of each estimate. Blue dots represent the point estimate of OR, and the horizontal lines depict the 95% CI.

In the recent 4 weeks, participants who engaged in heavy DIY activities, including tasks such as weeding, lawn mowing, carpentry, and digging, were evaluated for their risk of psychiatric diseases. Our Mendelian randomization analysis revealed a significant inverse association between these activities and the onset of psychiatric disorders (IVW: OR = 0.228, 95% CI: 0.113–0.461, *P* = 0.000038). Notably, both the QMR-Egger and horizontal pleiotropy tests returned *P*-values >0.05, indicating no evidence of horizontal pleiotropy. The QIVW test further confirmed the absence of heterogeneity with a *P*-value exceeding 0.05. Moreover, the leave-one-out analysis did not observe any significant shifts in the causal relationship, underscoring the reliability and robustness of our findings. The detailed outcomes of this association are illustrated in Figure 2 and summarized in Table 2.

**Table 2:** Association between different physical activity exposures and psychiatric diseases.

Exposure	Outcomes	Cochran *Q*-test				MR-Egger	
		QMR Egger	*P*	QIVW	*P*	Intercept	*P*
Heavy DIY Light DIY Strenuous sports aerobic exercises		14.0	0.447	14.8	0.466	−0.013	0.399
		6.32	0.611	6.73	0.665	0.008	0.539
	Psychiatric diseases	9.51	0.023	11.15	0.025	0.033	0.523
		12.7	0.242	12.8	0.308	−0.007	0.777

This table presents the Mendelian randomization analysis results assessing the causal effects of different types of physical activity exposures on psychiatric diseases. The analyses utilized QMR Egger, Cochran *Q*-test, and MR-Egger Intercept. The *P*-values listed help determine the statistical significance of each association.

### 3.3 Light DIY activities: implications for psychiatric disorders

We further explored the potential relationship between light DIY activities, such as pruning and watering the lawn, and psychiatric disorders. Our findings did not suggest a causal link between these activities and psychiatric disorders (IVW: OR = 0.918, 95% CI = 0.417–2.021, *P* = 0.831). Heterogeneity and horizontal pleiotropy tests consistently showed *P*-values >0.05. Additionally, the leave-one-out analysis did not detect any significant shifts in the causal relationship, emphasizing the consistency and robustness of our results. Comprehensive results are depicted in Figure 2 and Table 2.

### 3.4 Strenuous sports activities: a perspective on psychiatric disorders

The association between engaging in strenuous sports and the risk of psychiatric disorders was also examined. Our analysis did not identify a significant causal relationship (IVW: OR = 2.705, 95% CI = 0.081–3.419, *P* = 0.578). However, the presence of heterogeneity was indicated by the QIVW test with a *P*-value <0.05, prompting us to adopt a random-effects model for the Mendelian randomization analysis. The leave-one-out analysis remained consistent, with no notable variations in the causal relationship observed. The detailed findings are presented in Figure 2 and Table 2.

### 3.5 Aerobic exercises and their implications for psychiatric disorders

**Figure 3:**
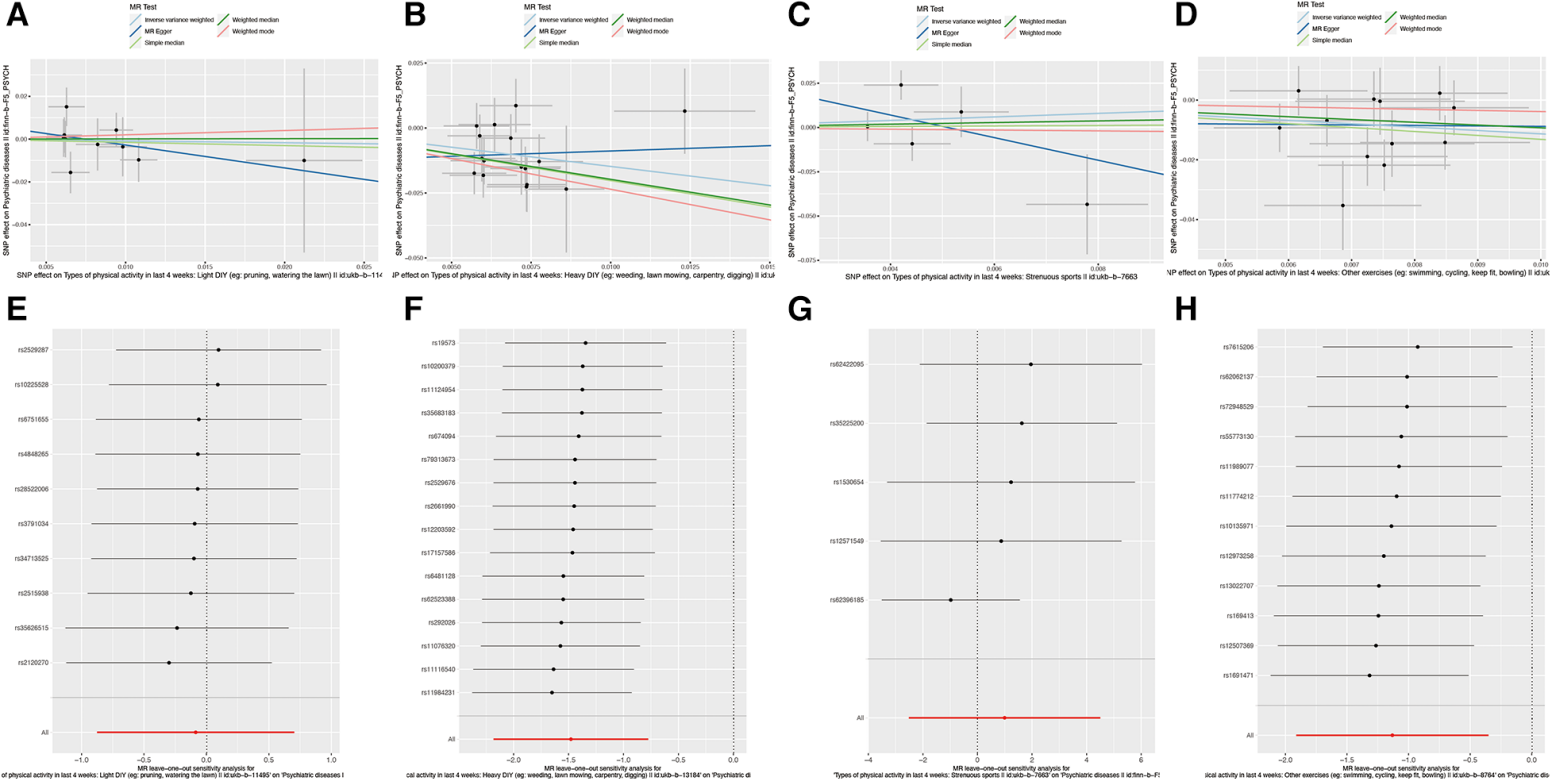
Causal relationship between SNP effects on various physical activities and the corresponding outcomes. (**A**) Scatter plot of the causal relationship between SNP effects and Types of physical activity in last 4 weeks: Light DIY (e.g., painting, watering the lawn). The slope of each line represents the causal relationship of each method. (**B**) Scatter plot of the causal relationship between SNP effects and Types of physical activity in last 4 weeks: Heavy DIY (e.g., weeding, lawn mowing, carpentry, digging). The slope of each line indicates the causal relationship determined by each method. (**C**) Scatter plot of the causal relationship between SNP effects and Types of physical activity in last 4 weeks: Strenuous sports. Each line's slope signifies the causal relationship based on the respective method. (**D**) Leave-one-out plot of the causal relationship between SNP effects and Types of physical activity in last 4 weeks: Light DIY (e.g., painting, watering the lawn). (**E**) Leave-one-out plot of the causal relationship between SNP effects and Types of physical activity in last 4 weeks: Heavy DIY (e.g., weeding, lawn mowing, carpentry, digging). (**F**) Leave-one-out plot of the causal relationship between SNP effects and Types of physical activity in last 4 weeks: Strenuous sports. (**G**) Leave-one-out plot of the causal relationship between SNP effects and Types of physical activity in last 4 weeks: Other exercises (e.g., swimming, cycling, keep fit, bowling).

Lastly, we assessed the potential impact of aerobic exercises, such as swimming, cycling, fitness training, and bowling, on psychiatric disorders. Our Mendelian randomization analysis suggested a possible causal association (IVW: OR = 0.322, 95% CI = 0.148–0.704, *P* = 0.004). The QIVW test indicated heterogeneity with a *P*-value <0.05, leading to the application of a random-effects model. Furthermore, the leave-one-out analysis did not identify any significant variations in the causal relationship, reinforcing the reliability and robustness of our results. The comprehensive outcomes of this association are showcased in Figure 3.

## 4 Discussion

Overall, our Mendelian Randomization (MR) study systematically identified types of physical activities that could play a role in the etiology of PD, offering potential guidance for physical therapy for PD. Specifically, our findings suggest a causal link between engaging in heavy DIY activities in the past 4 weeks and the onset of psychiatric diseases (IVW: OR = 0.228, 95% CI: 0.113–0.461, *P* = 0.000). Our analysis reveals that not all physical activities exert the same influence on mental health. Specifically, activities like swimming, cycling, fitness, and bowling appear to offer unique benefits. Swimming and cycling, for instance, may enhance mental well-being through cardiovascular improvements and stress reduction, while fitness activities and bowling can foster social interaction and mental engagement. These activities' varying intensities and social components may explain their differential impacts, suggesting a tailored approach to physical activity recommendations for psychiatric disease prevention and management. Additionally, when analyzing specific exercises, we observed a potential causal association between swimming, cycling, fitness, and bowling with psychiatric diseases (IVW: OR = 0.322, 95% CI: 0.148–0.704, *P* = 0.004). Surprisingly, our study did not establish a causal relationship between light DIY and psychiatric diseases (IVW: OR= 0.918, 95% CI: 0.417–2.021, *P* = 0.831) or between strenuous sports and psychiatric diseases (IVW: OR= 2.705, 95% CI: 0.081–3.419, *P* = 0.578). These results further underscore the hypothesis that not all exercises or any exercise intensity can yield benefits for PD.

Though the benefits of physical training for PD are well-known, such as how cardiovascular health largely impacts mental health (19), a previous meta-analysis indicated (20) a significant disparity between self-reported physical activity and objectively measured physical activity from patients with depression. This discrepancy may be influenced by mood states and cognitive biases, which can also impact mental health. Therefore, it's challenging to determine if the observed associations are genuine or merely a reflection of individual mood behaviors. For example, individuals predisposed to depression may perceive themselves as less active or engaged than their peers or may over report activities as compensation (21). While this doesn't invalidate the utility of self-report measures, it's crucial to validate its conclusions with objective measures. Hence, employing objective tools like SNPs to explore the genuine relationship between exercise and PD becomes highly appropriate.

The outcomes of randomized exercise trials, while generally favorable, are varied (22–24). In this study, we first determined that only when physical activity reached a certain intensity did it start to exert protective effects on PD. This suggests that light DIY activities, albeit beneficial in some respects, seem insufficient in offering protective or intervention effects for PD. This is consistent with some prior research conclusions suggesting that during the treatment of PD, emphasis should be placed on the dose of exercise (25). Perhaps moderate-intensity activities are more conducive to alleviating PD symptoms (26). On the other hand, high-intensity or competitive physical exercises often induce uncertain or detrimental physiological effects (11, 27, 28), emphasizing the need for appropriate dosage restrictions in therapeutic interventions. Our findings support this notion, as strenuous exercises appear unrelated to PD causally. Currently, a substantial body of evidence points towards selecting moderate-intensity physical and aerobic exercises for PD intervention, aligning with our conclusions (29, 30). Light DIY activities and strenuous exercises both seem ineffective for PD, whereas aerobic activities like swimming, cycling, fitness, and bowling yield evident benefits.

The impact of physical activity on PD can be multifaceted. Firstly, exercise affects the endocrine system and neurotransmitters, potentially explaining its effect on PD. Emphasis should be placed on how exercise training regulates stress responses through the hypothalamus-pituitary-adrenal (HPA) axis or glucocorticoid circulation (31, 32). To further elucidate the biological mechanisms through which physical activity exerts its beneficial effects on psychiatric disorders, recent research underscores the role of neuroplasticity and neurogenesis. Engaging in regular physical activity has been shown to enhance the expression of Brain-Derived Neurotrophic Factor (BDNF), a pivotal element in supporting neuroplasticity and the growth of new neural connections. BDNF not only aids in the repair and protection of brain cells but also plays a crucial role in mood regulation and cognitive functions. Moreover, physical activity's influence on neurogenesis, particularly in the hippocampus, contributes to improved emotional regulation and stress resilience. These enhancements in brain structure and function are key mechanisms by which exercise promotes mental health, highlighting the importance of physical activity as a complementary approach in the treatment and prevention of psychiatric disorders. For instance, physical exercise has been shown to reduce cortisol levels in patients with severe depression, which is considerably influenced by the type of exercise and its weekly frequency (33). Physical activity (PA) might alleviate symptoms through its potential impact on the inflammatory system, which is related to the etiology and severity of anxiety disorders. Moreover, functions in monoamine or endogenous opioid systems are also considered among the mechanisms of exercise interventions on mental health (9, 34). Inflammation is a critical element in the pathogenesis of psychological disorders, with elevated C-reactive protein levels associated with anxiety and depression (35). Persistent dysfunctions in the HPA axis may impair anti-inflammatory responses to glucocorticoids, leading to further inflammation (36). Exercise training, having anti-inflammatory properties, could positively affect anxiety treatment by mediating inflammatory pathways (37).

Furthermore, physical activity impacts intracranial tissue structures and functions (38). A reduced hippocampal volume, for instance, is linked with memory deficits in schizophrenia (39). Aerobic exercises have been proven to enhance the hippocampal volume, N-acetylaspartate/creatinine ratio, improve episodic memory in schizophrenic patients, and working memory in patients with major depression (40–42). Physical training can upregulate growth factors, like Brain-Derived Neurotrophic Factor (BDNF), stimulate neurogenesis and angiogenesis, facilitating functions of brain regions particularly related to anxiety or stress, aiding in reducing anxiety symptoms (43). Additional mechanisms encompass the impact of exercise interventions on the behavioral mechanisms of the psychological system, involving regulation (44) (e.g., tolerance and arousal regulation) and cognition (45, 46) (e.g., applying cognitive control over behaviors, maintaining attention, and flexibly shifting attention and behavioral responses to meet environmental demands), which might directly affect mental health (47), enhancing self-efficacy. Exercise can also boost reward significance by increasing participation in personally meaningful or beneficial activities and enhancing health perceptions (e.g., feeling healthier, improving body image) (12).

However, it's essential to recognize the significant limitations of this study. Primarily, the psychiatric diseases included in this research are based on all patients diagnosed with mental disorders according to ICD diagnostic codes. These cover a vast range of mental disorders, and their pathogenic mechanisms are not universally consistent. This might have caused an oversight in our study concerning specific disease types. Future research should be conducted separately for different kinds of psychological disorders. Secondly, there's an underrepresentation of non-European ancestries. Our study's focus on European populations introduces limitations in the generalizability of our findings to other ethnic groups. The genetic, cultural, and environmental factors that influence psychiatric disorders may vary significantly across different populations, potentially affecting the observed relationships between physical activity and psychiatric health. To address this limitation, future research should aim to include more ethnically diverse samples. Expanding the study to include populations from various backgrounds will help determine whether the protective effects of physical activities like swimming, cycling, fitness, and bowling are consistent across different ethnicities. This inclusivity will enhance our understanding of physical activity's role in psychiatric health and aid in developing more universally applicable therapeutic recommendations. Still, this doesn't imply that treatments can't benefit non-European ancestries. In the future, we should further expand our sample size to judiciously determine the most suitable exercise training mode and dosage for PD.

## 5 Conclusion

We've identified a potential causal link between engaging in extensive DIY physical activities or aerobic training (such as swimming, cycling, fitness, and bowling) and psychiatric diseases. However, no definitive causal relationship was established between light DIY or strenuous exercises and psychiatric disorders. Therefore, choosing the right type and intensity of exercise is crucial for psychiatric disease intervention.

## Data Availability

The original contributions presented in the study are included in the article/Supplementary Material, further inquiries can be directed to the corresponding author.
